# Complete genome sequence of psychrotolerant *Lacisediminihabitans* sp. FW035 isolated from freshwater in Antarctica

**DOI:** 10.1186/s12863-026-01465-z

**Published:** 2026-07-25

**Authors:** Minkyung Kim, Ahnna Cho, Minjeong Kwon, Yong-Joon Cho, Ok-Sun Kim

**Affiliations:** 1 Division of Life Sciences, Korea Polar Research Institute, Incheon, 21990 Republic of Korea; 2 Research Center for Endangered Species, National Institute of Ecology, Yeongyang, 36531 Republic of Korea; 3 Department of Molecular Bioscience, Kangwon National University, Chuncheon, 24341 Republic of Korea; 4 Multidimensional Genomics Research Center, Kangwon National University, Chuncheon, 24341 Republic of Korea

**Keywords:** *Microbacteriaceae*, *Lacisediminihabitans*, Antarctica, Biosynthetic gene cluster, isoprenoid

## Abstract

**Objectives:**

Microorganisms inhabiting cold and oligotrophic aquatic environments experience persistent physiological stress, necessitating genomic characterization to understand their survival strategies. Psychrotolerant strains have evolved diverse metabolic adaptations, including secondary metabolite biosynthesis, which may contribute to environmental fitness and offer potential for low-temperature biotechnological applications. However, the genus *Lacisediminihabitans* remains poorly represented at the genomic level, limiting our understanding of its ecological roles and metabolic potential. To address this gap, we generated a high-quality complete genome of a psychrotolerant *Lacisediminihabitans* strain isolated from Antarctic freshwater.

**Data description:**

*Lacisediminihabitans* sp. FW035 grew at 2 − 25 °C with an optimum at 20 °C. The genome of strain FW035 is 3,842,169 bp in size with a G+C content of 66.4%, encoding 3,592 protein-coding genes. Genome analysis revealed a complete C5–C20 isoprenoid biosynthesis pathway and multiple biosynthetic gene clusters, including terpene-associated clusters with low similarity to previously characterized pathways. These features indicate the presence of biosynthetic potential distinct from previously characterized pathways, particularly terpene-associated clusters, and highlight the dataset as a resource for future comparative genomic and functional analyses.

## Objective

Antarctica is one of the most extreme environments on Earth, characterized by persistently low temperatures, strong freeze–thaw cycles [1], oligotrophic conditions [2], and high levels of ultraviolet radiation [3]. Microorganisms inhabiting these environments must therefore rely on specialized metabolic and physiological strategies to withstand oxidative stress, nutrient limitation, and strong temperature fluctuations [4]. These ecological pressures make Antarctic freshwater bacteria valuable model systems for studying microbial adaptation and metabolic versatility, with potential relevance for low-temperature biotechnological applications [5].

The genus *Lacisediminihabitans* was first proposed in 2020 as a new member of the family *Microbacteriaceae* in the phylum *Actinomycetota* based on genotypic and phenotypic analyses [6]. According to the List of Prokaryotic names with Standing in Nomenclature (LPSN) database, only two type strains of *Lacisediminihabitans profunda* and *Lacisediminihabitans changchengi* have been validly published to date (https://lpsn.dsmz.de/search?word=lacisediminihabitans). Both strains are psychrotolerant, yet currently available genome assemblies remain limited and incomplete. The limited number of available strains hampers comprehensive exploration of the ecological adaptations and genomic diversity of this genus, thereby highlighting the importance of genome-based characterization of additional *Lacisediminihabitans* strains.

To address this gap, a psychrotolerant strain, *Lacisediminihabitans* sp. FW035, was isolated from Antarctic freshwater and subjected to whole-genome sequencing. The objective of this study was to generate a high-quality complete genome dataset to support taxonomic identification, comparative genomics, and functional annotation of this poorly characterized genus.

## Data description

*Lacisediminihabitans* sp. FW035, which was tested for growth at at 2–37 °C and exhibited growth between 2 and 25 °C (optimum, 20°C), was cultivated on R2A medium at 20 °C. Genomic DNA was extracted using a FastDNA spin kit (MP Biomedicals, USA) according to the manufacturer’s instructions. Whole-genome sequencing was performed using Illumina NovaSeq and Oxford Nanopore platforms. For Illumina sequencing, 50 ng of genomic DNA was processed with the NEBNext® Ultra™ II DNA Library Prep Kit and sequenced at AZENTA (USA) on the NovaSeq 6000 system (Illumina, USA) using a 2 × 100 bp paired-end configuration. The Illumina raw data were adapter- and quality-trimmed using BBDuk (v39.06) [7] and deposited under SRA accession SRR37367711 (Table 1, Data set 1). Nanopore sequencing was performed on a MinION Mk1B (Oxford Nanopore Technologies, UK). Nanopore reads were trimmed in MinKNow (Table 1, Data set 2), assembled using Flye (v2.9.4-b1799) [19], and the resulting contig was polished with quality-trimmed Illumina reads using Polypolish (v0.6.0) [20]. Default parameters were used except where otherwise noted.

**Table 1:** Overview of data files/data sets

Label	Name of data file/data set	File types (file extension)	Repository and identifier (DOI or accession number)
Data set 1	Paired-end Illumina raw reads of *Lacisediminihabitans* sp. FW035	Sequence file (.fastq.gz)	Sequence Read Archive (https://identifiers.org/ncbi/insdc.sra:SRR37367711) [8]
Data set 2	Oxford Nanopore raw reads of *Lacisediminihabitans* sp. FW035	Sequence file (.fastq.gz)	Sequence Read Archive (https://identifiers.org/ncbi/insdc.sra:SRR37367710) [9]
Data set 3	Complete genome sequence of *Lacisediminihabitans* sp. FW035	Sequence file (.fasta)	Nucleotide Sequence Database (https://identifiers.org/ncbi/insdc:JBTZBY000000000) [10]
Data file 1	Circular genome map of *Lacisediminihabitans* sp. FW035	PDF file (.pdf)	Figshare (https://doi.org/10.6084/m9.figshare.31889905) [11]
Data file 2	General and genomic features of *Lacisediminihabitans* sp. FW035	MS Excel file (.xlsx)	Figshare (https://doi.org/10.6084/m9.figshare.31897243) [12]
Data file 3	16S rRNA gene-based phylogenetic tree	PDF file (.pdf)	Figshare (https://doi.org/10.6084/m9.figshare.31889947) [13]
Data file 4	Genome-based phylogenetic tree	PDF file (.pdf)	Figshare (https://doi.org/10.6084/m9.figshare.31889998) [14]
Data file 5	ANI, AAI, and dDDH comparison results	MS Excel file (.xlsx)	Figshare (https://doi.org/10.6084/m9.figshare.31890070) [15]
Data file 6	COG functional classification	PDF file (.pdf)	Figshare (https://doi.org/10.6084/m9.figshare.31896106) [16]
Data file 7	KEGG annotation results	PDF file (.pdf)	Figshare (https://doi.org/10.6084/m9.figshare.31896115) [17]
Data file 8	Biosynthetic gene cluster (antiSMASH) results	MS Excel file (.xlsx)	Figshare (https://doi.org/10.6084/m9.figshare.31896136) [18]

The complete genome comprises a single circular chromosome of 3,842,169 bp with a GC content of 66.4% (Table 1, Data set 3 and Data file 1). It contains 3,592 protein-coding genes, three rRNA genes (5S, 16S, and 23S rRNA genes), and 50 tRNA genes (Table 1, Data file 2). Quality analysis was performed using CheckM (v1.2.4) [21], indicating 98.78% completeness and 0.79% contamination. The taxonomic classification based on 16S rRNA genes showed that strain FW035 clustered closely with *L. profunda* CHu50b-6–2 (98.82% similarity) (Table 1, Data file 3). In the genome-based phylogenomic tree, strain FW035 was also placed within the genus *Lacisediminihabitans* (Table 1, Data file 4), further supported by a POCP of 67.31% with *L. profunda* CHu50b-6–2. Comparative genomic analyses indicate that strain FW035 represents a genomically distinct lineage within the genus *Lacisediminihabitans* based on ANI, AAI, and dDDH values (ANI, 77.8%; AAI, 73.5%; dDDH, 21.3%) (Table 1, Data file 5).

COG and KEGG annotations revealed a functional repertoire consistent with heterotrophic metabolism, including carbohydrate and amino acid transport and metabolism (Table 1, Data set 6 and 7). Genome mining using antiSMASH (v8.0) [22] identified six biosynthetic gene clusters (BGCs), including terpene, type III polyketide synthase, lanthipeptide class V, non-α poly-amino acid, and β-lactone-associated clusters (Table 1, Data file 8). Only one cluster showed high similarity (≥75%) to a known BGC, while the remaining clusters exhibited low similarity (<15%) to previously characterized clusters. Strain FW035 harbors genes involved in terpene synthesis with only low similarity to characterized carotenoid-like clusters. These findings suggest that strain FW035 harbors biosynthetic potential distinct from previously characterized clusters, representing candidates for future functional characterization, particularly the terpene-associated BGCs with low similarity to known carotenoid pathways. KEGG pathway annotation indicated the presence of genes comprising a complete C5–C20 isoprenoid backbone biosynthesis pathway, including genes associated with the synthesis of isopentenyl diphosphate and dimethylallyl diphosphate, and downstream prenyl diphosphates (geranyl-, farnesyl-, and geranylgeranyl-diphosphates). The coexistence of a complete isoprenoid pathway and multiple terpene-associated BGCs suggests that strain FW035 may contribute to oxidative stress mitigation and membrane stabilization through terpenoid-derived compounds, potentially enhancing ecological fitness under the high UV irradiance and oligotrophic conditions characteristic of Antarctic freshwater habitats.

## Limitations

The dataset provides the complete genome of a *Lacisediminihabitans* strain; however, no experimental validation was performed to confirm the functional activity of the predicted genes or biosynthetic pathways. The presence of biosynthetic gene clusters does not confirm the production of corresponding metabolites under natural or laboratory conditions. Functional annotations are based on computational predictions and available databases, which may include incomplete or inaccurate assignments.

## Data Availability

Strain FW035 has been deposited in the Korean Collection for Type Cultures (KCTC) under the accession number KCTC 59629. The complete genome assembly of *Lacisediminihabitans* sp. FW035 has been deposited in GenBank under accession number JBTZBY000000000 with BioProject PRJNA1354626 and BioSample SAMN52950395. Illumina and Oxford Nanopore raw reads are available in the Sequence Read Archive (SRA) under accession numbers SRR37367711 and SRR37367710, respectively. Please see Table 1 and references [8–10] for details and links to the data.

## Abbreviations

LPSN: List of Prokaryotic names with Standing in Nomenclature
POCP: Percentage of conserved proteins
ANI: Average Nucleotide Identity
AAI: Average Amino acid Identity
dDDH: Digital DNA–DNA Hybridization
COG: Clusters of Orthologous Genes
KEGG: Kyoto Encyclopedia of Genes and Genomes
BGCs: Biosynthetic Gene Clusters
